# When Does Maluma/Takete Fail? Two Key Failures and a Meta-Analysis Suggest That Phonology and Phonotactics Matter

**DOI:** 10.1177/2041669517724807

**Published:** 2017-08-25

**Authors:** Suzy J. Styles, Lauren Gawne

**Affiliations:** Division of Psychology, Nanyang Technological University, Singapore; 4913 SOAS, University of London, England; La Trobe University, Australia

**Keywords:** cross-cultural perception, cross-modal congruence, language-specific perception, pseudoword legality, sound symbolism

## Abstract

Eighty-seven years ago, Köhler reported that the majority of students picked the same answer in a quiz: Which novel word form (‘maluma’ or ‘takete’) went best with which abstract line drawing (one curved, one angular). Others have consistently shown the effect in a variety of contexts, with only one reported failure by Rogers and Ross. In the spirit of transparency, we report our own failure in the same journal. In our study, speakers of Syuba, from the Himalaya in Nepal, do not show a preference when matching word forms ‘kiki’ and ‘bubu’ to spiky versus curvy shapes. We conducted a meta-analysis of previous studies to investigate the relationship between pseudoword legality and task effects. Our combined analyses suggest a common source for both of the failures: ‘wordiness’ – We believe these tests fail when the test words do not behave according to the sound structure of the target language.

## Introduction

Demonstrations of Köhler’s maluma/takete effect (1929/1947) have continued over several decades, in a variety of contexts (for recent review, see Lockwood & Dingemanse, 2015) including recent replications in remote groups, such as the Otjiherero-speaking Himba participants from Namibia (Bremner et al., 2013). Many researchers now tacitly agree the effect may have universal sensory underpinnings (Imai & Kita, 2014; Maurer, 1993; Maurer, Pathman, & Mondloch, 2006; Ramachandran & Hubbard, 2001). However, in an often overlooked report, Rogers and Ross (1975) failed to find the effect while conducting fieldwork in Papua New Guinea: Twenty participants identified as Songe (speakers of a subdialect of Hunjara, a language of the Orokaiva group) were split between expected and unexpected responses (9 to 11). Since their report, no other failures have seen the light of day, using either the original maluma/takete word forms or other variants (e.g., bouba/kiki: Ramachandran & Hubbard, 2001). It has therefore remained a mystery when, and under what circumstances, these effects differ between groups. Fortunately, our own recent failure represents a timely opportunity to revisit conditions that generate sound symbolic failures.

## Study 1: Behavioural Test

### Method

Syuba (also known as Kagate, ISO 639-3 syw) is a Central Bodish language of the Tibeto-Burman family, with a population of around 1,500 speakers (Gawne, 2013). It is one of a number of mutually intelligible Yolmo (ISO 639-3 scp) dialects, which share lexical tone: a high-versus-low tone contrast on the initial-syllable (Teo, Gawne, & Baese-Berk, 2015). Yolmo speakers traditionally live in the higher hills and mountains of the Himalaya (see Gawne, 2016 for further details). Syuba speakers are all multilingual in Nepali for trading, with younger speakers undertaking formal education in Nepali and English (Mitchell & Eichentopf, 2013).

The second author recorded a native speaker of Lamjung Yolmo (a mutually intelligible Yolmo variety, selected so his voice would be unfamiliar to participants) in a quiet room in Kathmandu, saying ‘kiki’ and ‘bubu’ several times, until a clear version was recorded without noise from local traffic, chickens and so forth. In Yolmo, including Syuba, words starting with /k^h^/ take high tone, and /b/ low tone. As /k^h^/ only occurs word initial, and always co-occurs with tone, the production of ‘kiki’ /k^h^ík^h^í/ resulted in HH, and by analogy the speaker produced ‘bubu’ /bùbù/ with LL. The use of reduplicated syllables (cf., Ozturk, Krehm, & Vouloumanos, 2013) generated consistent tone within each word form, even though Yolmo tones usually even out after the first syllable (Teo et al., 2015). Since higher pitches “go with” more angular shapes, and vice versa (e.g., Walker et al., 2010), we expected the Syuba tones to enhance the spikiness of ‘kiki’ and the blobbiness of ‘bubu.’ The recorded tokens are available in the Open Science Framework repository for this article (Gawne & Styles, 2017: https://osf.io/wt95v/).

For visual stimuli, we chose a spiky and a curvy shape, which have previously been shown to elicit matches to ‘kiki’ and ‘bubu’ word forms for South East Asian participants (Hung, Styles, & Hsieh, 2017). Since not all of our Syuba speakers would be familiar with paper-based representations, we decided to present the stimuli as physical objects instead. To generate travel-hardy objects, templates were traced out and cut from a sheet of firm, but flexible craft plastic. Both objects were covered in a self-adhesive layer of felt, in a neutral tone, to prevent injury from the sharp point of the kiki spikes.

**Figure 1.**
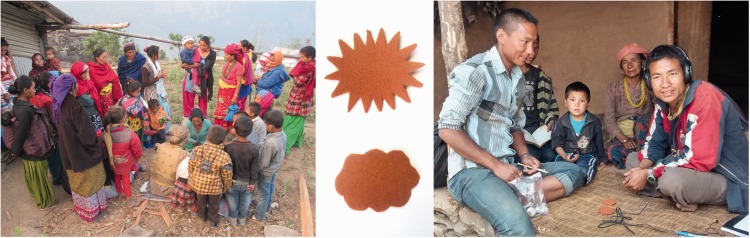
Syuba-speaking participants making kiki/bubu decisions in Nepal, with photographs of the physical object tokens used in decision-making.

### Results and Discussion

Out of 24 Syuba participants, the shape-selection task was completed in the following ratio: congruent-to-incongruent, 11 to 13 – A resounding failure.^1^ Since this is only the second documented case of healthy, typically developing people failing to show this well-documented effect, we believe that unpacking the source of this failure can provide a more nuanced understanding of the effect itself. In the following section, we present a linguistic analysis of underlying biases in canonical sound-symbolism test items, and how these biases may be able to explain the failure of both Rogers and Ross’s (1975) Songe participants, and our Syuba participants.

## Study 2: Linguistic Analyses

**Figure 2.**
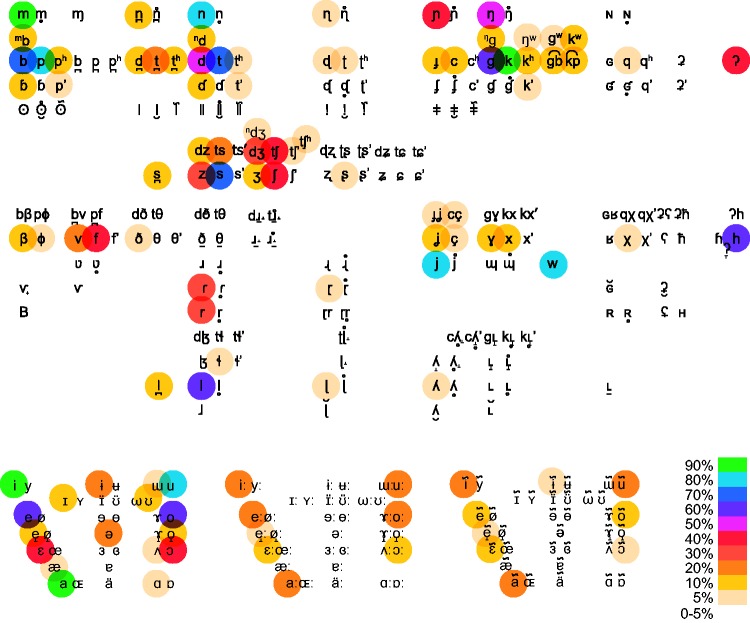
Prevalence rates of speech-sounds across 1,672 languages. Data from PHOIBLE Online. Colour scale indicates range from the listed percentage to the next highest. For data tables underlying this figure, see the Open Science Framework repository for this article (https://osf.io/wt95v/).

Figure 2 presents a visual array of prevalence rates for consonants and vowels across different languages. The figure is an extended International Phonetic Alphabet chart overlayed with colours showing the percentage of languages reported to include each sound. The data are drawn from the PHOIBLE Online database of 2,160 segments from 1,672 documented languages (Moran, McCloy, & Wright, 2014). While by no means an exhaustive listing of all sounds from all languages of the world, this visualization illustrates a general principle of linguistic typology: Some sounds turn up in more languages than others. It should be noted that the distribution of sounds across languages follows something like Zipf’s Law (Zipf, 1935), with few sounds occurring in almost all languages, and the majority of sounds occurring in very few languages, as can be seen in the number of uncoloured segments (indicating that they occur in less than 5% of languages in the PHOIBLE data set).

Highly prevalent sounds (occurring in >50% of languages) include the three peripheral vowels /i/ /a/ /u/, followed by two mid-high vowels /e/ /o/, and among consonants, nasals and obstruents at three places of articulation (bilabial: /p//b//m/, alveolar: /t//d//n/, velar: /k//g//ŋ/), along with the voiceless velar fricative /h/, two semivowels /w/ /j/, a voiceless sibilant /s/, and a liquid /l/. Notably, all of these sounds occur in English and the majority of common European languages.

Keen-eyed sound-symbolism researchers will notice that these highest prevalence sounds are also the sounds most commonly investigated in tests of the maluma/takete effect. This trend is particularly clear in studies that involve matching shapes to invented pseudowords, where stimuli are almost exclusively constructed from /p//b//m/, /t//d//n/, /k//g/ and /i//e//a//o//u/ (e.g., see D'Onofrio, 2014; Fort, Martin, & Peperkamp, 2015; Nielsen & Rendall, 2011, 2013; Westbury, 2005).

The two most commonly tested linguistic pseudowords are maluma/takete (the original test pair, developed by Kohler) and bouba/kiki (developed by Ramachandran and Hubbard). Table 1 shows the segment-by-segment prevalence rates for each of the phonemes in the canonical words, along with the mean prevalence rates for sounds included in each word pair (Moran et al., 2014). Both word pairs are comprised exclusively of sounds with high prevalence rates (above 50%).

**Table 1.** Prevalence Rates of Phonemes in Canonical Test Words.

Rounded						Angular					
	/b/	/u/	/b/	/a/			/k/	/i/	/k/	/i/	
	71%	87%	71%	91%			94%	93%	94%	93%	
/m/	/a/	/l/	/u/	/m/	/a/	/t/	/a/	/k/	/e/	/t/	/e/
95%	91%	66%	87%	95%	91%	74%	91%	94%	68%	74%	68%

Why should ‘canonical’ test words be constructed of such high-prevalence phones to begin with? One likely explanation is that both human language and linguistic sound symbolism benefit from discriminability, or “perceptual separation” (cf., Ladefoged, 1993), which can be achieved easily when differences in the vocal tract are used to maximize acoustic contrasts. For example, among consonants, voiced versus voiceless, sonorant versus abrupt sounds made at the front versus the back of the mouth give highly contrastive acoustic profiles (e.g., /m/ vs /k/, respectively). Almost all languages use this contrast in their phomenic inventories (>90%). Given their perceptual discriminability, it is perhaps hardly surprising that these tokens act as exemplary “end points” in cross-modal mappings between speech sounds and other graded perceptual stimuli, such as shape (i.e., curvy, spiky). Similar perceptual linkages have been observed in the extensive literature on cross-modal correspondences between graded perceptual spectra including pitch, brightness, height, size and loudness (cf., Spence, 2011), some of which can be observed in infancy (Walker et al., 2010) and in nonhuman primates (Ludwig, Adachi, & Matsuzawa, 2011).

However, not all sounds that are highly discriminable *exist* in all languages, and this mismatch can lead to unintended sources of bias in experimental stimulus sets (a point that has been made elsewhere by Styles, 2014). First, sounds that are within the experimenter’s language may be assumed to be more ‘universal’ than they really are (a mismatch of local-to-global prevalence). For example, English sounds like the fricatives /f//v/ and the approximant /ɹ/ have relatively low global prevalence (49%, 29% and less than 5%, respectively), and their inclusion in cross-linguistic stimuli may compromise or complicate data collection. Second, for a supposedly ‘universal’ effect, we know nothing about the sound-symbolic properties of low-prevalence sounds like ejectives /b’/, ingressives /ɓ/ and obstruents at retroflex /ʈ//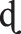/ and uvular /q//G/ places of articulation, and whether their cross-modal matching would differ between groups of participants who have different experience with these kinds of sounds. Both of these problems fit the model of Western, Educated, Industrialized, Rich, and Democratic (WEIRD) biases (Henrich, Heine, & Norenzayan, 2010), according to which the majority of scientific evidence comes from a small subset of people from predominantly WEIRD nations, and hence our assumptions about what is ‘normal’ often come from our own experience of the world. A related source of bias is that even when we do try to select sounds for their ‘universality,’ they may not actually occur in a particular language (a mismatch from global-to-local prevalence). For this reason, a more thorough consideration of stimulus items is needed, both to understand exactly what we have been measuring so far, and to unpack how it relates to the human experience more broadly.

An underlying assumption in linguistic sound symbolism is that humans and other animals share some degree of structural similarity in our multisensory processing systems, through either shared evolutionary heritage (Morton, 1977; Ohala, 1994) or shared sensory experience of the same environment (Maurer, 1993; Maurer et al., 2006; Mondloch & Maurer, 2004; Spence, 2011). If the sensory substrates of cross-modal matches are innately universal, then whether or not a particular sound exists in a particular speaker’s language would not be expected to influence sound-symbolic ‘matching.’ On the other hand, if these matching processes are subject to environmentally driven plasticity, then we would expect to see language-specific differences. Given the well-known phenomenon of phonological ‘tuning’ processes in infancy, and its outcomes on adult perception (Iverson & Kuhl, 1996; Kuhl, 2004, 2010; Polka & Bohn, 2003; Schwartz, Abry, Boë, Ménard, & Vallée, 2005; Werker & Tees, 1984), the presence or absence of a sound in a speaker’s language may indeed impact the perception of a cross-modal match.

Given these considerations, there is no guarantee of a match between the test word and a target language. To give an example, ‘bouba’ ‘kiki’ and ‘takete’ would be legitimate word forms in Japanese, but ‘maluma’ would fail, due to the absence of Japanese [l]. Similarly, ‘bouba,’ ‘kiki’ and ‘maluma’ are legitimate word forms in Tiwi (a language from the Tiwi islands, off the North coast of Australia), but this time, ‘takete’ would fail, due to the absence of Tiwi [e] (Moran et al., 2014). That is to say, for speakers of a given language, some of the test items are simply more ‘wordy’ than others. Even though the sounds in these words are high prevalence, more than 30% of the world’s languages would be missing one or more of these sounds. As such, the status of a test item as a possible word in the speaker’s language (the item’s ‘wordiness’) may influence when maluma/takete effects arise. With this in mind, we evaluate the status of the test words in two cases of failure: Songe (Hunjara) speakers (Rogers & Ross, 1975) and our own Syuba speakers.

### Match Between Failed Test Words and Target Languages

We conducted a check of the ‘wordiness’ of each stimulus item, against published descriptions of the sound structures of the languages. Table 2 presents a summary of mismatches between the target words and the languages’ acoustic structures. According to Gray, Hiley, and Thom’s recent documentation (2015), the Hunjara language of Papua New Guinea does not contain the sounds [l] or [t^h^]. This means that despite the familiarity of these sounds to English speakers, the ‘maluma’ and ‘takete’ test items used by Rogers and Ross (1975) were not a good match to the structural regularities of the Hunjara language. In the case of our participants, following analysis from Gawne (2013), although none of the test sounds were missing from Syuba phoneme inventory, *the combination of sounds* violated the sound patterns of Syuba words: While the [k^h^] does occur in Syuba, it never occurs word-medially, and while [u] occurs, it never appears at the end of a two-syllable word. Furthermore, given the unusual sounds in the second syllables of both words, our speaker effectively pronounced the tones in each word as though it was made of two first syllables, with reduplicated tones /HH/ and /LL/, rather than the tone levelling that normally occurs in long Syuba words (i.e., /H-neutral/ and /L-neutral/). Thus, we can conclude that Rogers and Ross used stimuli that violated the phonetic structure of the Hunjara language, and we used stimuli that violated the phonotactic and tonotactic structure of the Syuba language. In both cases, the test items were not ‘wordy’ in the target languages.

**Table 2.** When Maluma/Takete Fails.

	Hunjara (PNG)^a^		Syuba (Nepal)^b^	
Language	Regularity	Illegal item	Regularity	Illegal item
	No [l]	*maluma	No word-medial [k^h^] No 2-syl./HH/tone	*kiki *HH
	No [t^h^]	*takete	No 2-syl. word-final [u] No 2-syl./LL/tone	*bubu *LL
Reason	Phonetic violation		Phonotactic/tonotactic violation	

*Note*. PNG = Papua New Guinea.

a Phoneme inventory from Gray, Hiley, and Thom (2015).

b Phonological analysis from Gawne (2013).

In both the Syuba language reported here and the Hunjara language of Rogers and Ross (1975), the words used in the test did not match the sound structure of the target language. This suggests a possible link between the lack of ‘wordiness’ in a test stimulus and failure to show the expected maluma/takete effect.

## Study 3: Meta-Analysis of Published Maluma/Takete Effects

To put these two failures in context, we conducted a review of published maluma/takete effects. The purpose of the meta-analysis is to characterize previously published studies according to whether the stimuli should be considered ‘legal’ pseudowords for speakers of the target language and to use the multitude of published studies to establish the expected strength of maluma/takete effects for ‘legal’ pseudowords. Within this context, the meta-analysis allows us to investigate whether the data arising from these two ‘illegal’ pseudoword studies (i.e., nonwords) overlap with the former distribution. That is to say, whether these two small studies should be considered as statistically predictable outerliers in a wide general distribution, or whether they represent a separate, statistically discrete, distribution.

### Methods

We conducted a semi-systematic review of published articles containing some version of the bouba/kiki or maluma/takete test, implemented as a choice between two shapes differing in roundness/angularity, in response to auditory stimuli similar to the original word pairs. Further details of the method and the articles included in the study can be found in the Supplementary Materials for this article. Figure 1 summarizes data from studies in which participants heard an auditory pseudoword (or a pair of pseudowords) and selected a best match from a pair of pictures exhibiting a curvy/spiky shape difference using a binary match design, where the word forms were designed according to the CVCV(CV) style of maluma/takete-bouba/kiki pseudowords, and with *predominantly similar* phonological content (i.e., /m, l, b, u, o/ for rounded, and /k, t, i, e/ for angular).

Studies were included if:

- The test presented an auditory pseudoword or a pair of auditory pseudowords.
- The test included pictures exhibiting a curvy/spiky contrast.
- The test was implemented to generate a binary match: EITHER a choice between two pictures (a picture preference test), OR where ratings for pairs of stimuli were translated into a binary measure per individual (e.g., rating for match between ‘maluma’ and curvy shape was greater than match between ‘maluma’ and spiky shape).
- The report included the number of participants tested, for use in computing standard error, 95% confidence levels and weighting for individual studies (Ramachandran & Hubbard’s, 2005 “large classroom” was conservatively estimated as *N* = 100).
- Participants in a given group were neurotypical adolescents or adults.

Studies were excluded if:

- The test involved decisions about word ‘meaning’ rather than shape-matching (i.e., the substantial literature on ideophones expressing adjectival/adverbial properties, or names of nouns).
- The stimuli did not include canonical phonological structure (i.e., /m, b, l, u, o/ for rounded, and /k, t, i, e/ for angular) for the majority of consonants and the majority of vowels in the majority of words. For example, the recent study by Drijvers, Zaadnoordijk, and Dingemanse (2015) was excluded as the ‘pointy’ words did not include voiceless stop consonants.
- Noncanonical forms made up the majority of stimuli in a pooled data set and item-level statistics were not available in the published document.

#### Data inclusion/exclusion

- Where data were available for individual trials, each trial using canonical phonological stimuli was included in the meta-analysis (e.g., a ‘bouba’ trial and a ‘kiki’ trial in Bremner et al., 2013).
- Where data were available for multiple types of trials, trials were included if they used canonical phonological stimuli and were excluded if they included a mix (e.g., only Condition 1 from Nielsen & Rendall, 2011 was included), or used substantially different phonemes (e.g., only selected trial types from Fort et al., 2015).
- Where some subsets of the data came from clinical groups (Oberman & Ramachandran, 2008; Occelli, Esposito, Venuti, Arduino, & Zampini, 2013), only participants from the neurotypical control groups were included.

#### Pseudoword ‘legality’

- Pseudowords in all nine studies for English-speaking participants were classed as phonetically and phonotactically ‘legal’ as judged by the authors (both native English-speaking linguists).
- Pseudowords in one study with Italian-speaking participants (Occelli et al., 2013) were judged to be phonetically and phonotactically ‘legal’ by the first author of that study, who noted a small proportion of the pseudowords ended in consonants, which is a low-frequency word type in Italian, but none were nonwords (personal communication with the first author).
- Pseudowords in studies with French-speaking participants (Fort et al., 2015; Peiffer-Smadja, 2010) were judged as legal by two researchers with expertise in French phonology or phonetics, who noted that some pseudowords represented low frequency word types, but none were nonwords (personal communication with the second author).
- For one study with Himba participants who speak a dialect of Otjiherero (Bremner et al., 2013), a reference grammar was consulted (Möhlig, Marten, & Kavari, 2002). Vowels in the test words /buba/ and /kiki/ were attested in the vowel inventory. Among consonants, bilabial and velar places of articulation are both attested (/p, k/), but voiced/voiceless contrasts were not attested in the stop consonant inventory. This means the consonants in /buba/ and /kiki/ would exhibit a salient phonological contrast according to place (bilabial/velar), but no contrast due to voicing or aspiration. We have classed these data as phonologically ‘suspicious’ and moved them from the meta-analysis to the “comparison set” for comparison with published ‘legal’ studies and further discussion.

#### Data handling

- Data were included if they could be expressed as either the number of participants who gave the expected outcome along with the total number of participants, or participants’ mean response over multiple tests, along with the group standard error.
- In articles where means and standard errors were given in figures only, values were measured from the figures.
- Where multiple trials were performed by a single participant within a single study, the results were combined to produce a mean for that experiment.
- Where a single paper reports multiple experiments with different groups of participants, the experiments are treated as separate data sets, as they represent the responses of different people.

#### Meta-Analysis

The meta-analysis was conducted using the Random Effects Model described by Neyeloff, Fuchs, and Moreira (2012). This procedure allows calculation of values from studies with standard error arising from different computations (i.e., number of occurrences vs. mean of means). Standard error was as reported in the original article or calculated according to Hackshaw (2009). The data tables for the analysis can be found in the Supplementary Materials for this article, as well as in the Open Science Framework repository for this article (https://osf.io/wt95v/).

### Results and Discussion

**Figure 3.**
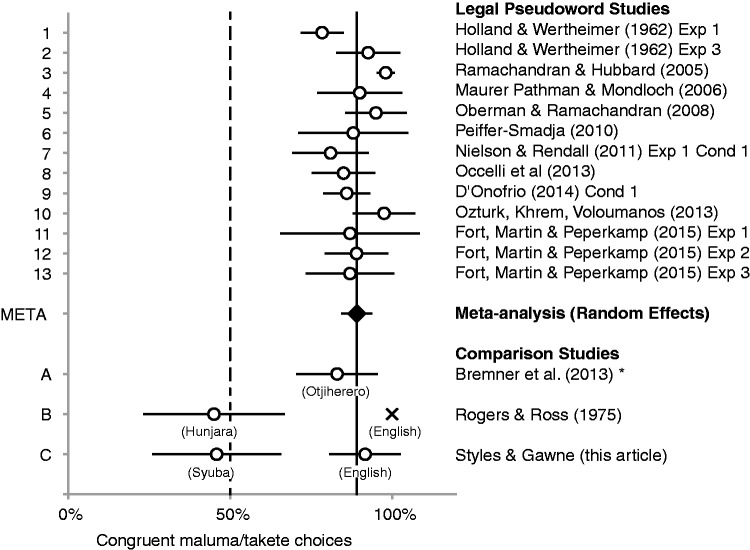
Forest plot of published maluma/takete effects for canonical stimuli. 1 to 13: Pseudowords “legal.” META: Results of the random effects meta-analysis. A to C: “legality” of pseudowords questionable for some groups of participants. *Participants articulated pseudoword before matching shape to word form. Chance response rate (50%) marked with dashed line. Horizontal bars show 95% CIs. X no *SE* available. For further details of the Forest Plot, see Supplementary Materials and the Open Science Framework repository for this article (https://osf.io/wt95v/).

The results of the meta-analysis are given in Figure 3, where each discrete data set is represented as a single row of data, where the marker represents the effect size, and the horizontal line, the 95% confidence interval (CI). Chance (50%) is marked with a dashed line, and the meta-analysis of all studies is represented by a diamond summarizing the data sets from the “Legal Pseudoword studies.” According to the results of the meta-analysis, published reports of a bouba/kiki test using predominantly *canonical* speech sounds with phonologically and phonetically *legal stimuli* report an average rate of 89% congruent responses in binary preference procedures (95% CIs [84, 94]). The observed power of this effect calculated in G*Power (Faul, Erdfelder, Lang, & Buchner, 2007) is effectively 1, meaning that the maluma/takete responding at above chance rates should be highly replicable, even with small samples, so long as the majority of the stimuli comprise of the canonical /b, m, l, o, u/-round, and /t, k, i, e/-spiky, phonemes.

Below the meta-analysis are data from studies where phonological ‘legality’ of the stimuli is less clear, for comparison with the meta-analytic average. In the Himba study with speakers of Otjiherero (Bremner et al., 2013), the data align well with the published pattern of bouba/kiki effects, as reported in the original article. By contrast, in the two studies of interest here, the response rates for non-English-speaking participants are substantially different. Both of these studies included an English language comparison group, where it is clear that the English-speaking controls performed in line with the published pattern, as shown by their degree of overlap with the results of the meta-analysis (although note that there was no sample size included in the report from Rogers & Ross, 1975). The lack of overlap between the 95% CIs of the two types of experiments shows that our Syuba data and Rogers and Ross’s Hunjara data represent a pattern of data discrete from the other data sets, effectively aligning with chance.

Following the meta-analysis, we conducted a further exploratory analysis (unplanned prior to data collection). We used the meta-analytic mean of 89% as a test value against which we compared the results of the two studies of interest, to check whether these samples are sufficiently well powered for comparison, using power analysis in G*Power (Faul et al., 2007). For Hunjara (*M* = 0.45, *SD* = 0.38), the effect size was large (*d* = 1.16), and the observed power was high (.9995), suggesting that the original sample size of 20 participants is sufficiently well powered, and for future replications seeking a minimum power of .95, as few as 10 participants should suffice if the effect size is representative of the general Hunjara-speaking population. For Syuba (*M* = 0.46, *SD* = 0.50), the effect size was also large (*d* = 0.86), and the observed power also high (.993), suggesting that the original sample size of 24 participants is sufficiently well powered, and that future replications with this group could be expected to differ from the previously reported mean with as few as 16 participants at a minimum Power of .95, if the effect size here is representative of the general Syuba population. These Power analyses suggest that although the sample sizes here are small, they are sufficiently well powered for comparison with the previously published pattern of responses. Notably, these are the two studies in which the pseudowords contained multiple deviations from the phonology or phonotactics of the participants’ languages and did not involve participants repeating the word prior to making a shape selection.

So, given that the test words used with Himba participants (Otjiherero) contained sounds not reported in the phonological inventory of their language, why did the Himba study align with the typical pattern but not the other two studies? One likely reason is perceptual assimilation: According to the Perceptual Assimilation Model of Best and colleagues, nonnative phones articulated at the same place using the same articulatory organs are perceptually assimilated to attested phonological targets (Best, McRoberts, & Goodell, 2001). Hence, even though /b/ does not have phonemic status in Otjiherero, [b] and [p] could be perceived as variants of the single bilabial category /p/. Furthermore, according to the description of the methods (Bremner et al., 2013), the instructions were verbally translated for the participant, and the participant was asked to articulate the test word before making their choice. It is therefore likely that any unfamiliar sounds in the experimenter’s utterances were assimilated into Otjiherero-compliant forms, either by the translator or by the participants themselves.

To give an example of this kind of assimilation during production, similar processes can be seen when native speakers of English and Japanese pronounce foreign loan words from each others’ languages: In standard Japanese, the combination /tu/ is unattested, so the English loan word ‘tuna’ becomes /tsuna/ in Japanese. By contrast in English, the combination /ts/ does not occur at the start of words, so the Japanese loan word ‘tsunami’ becomes /sunami/ in English. These kinds of changes represent assimilation of nonnative sound combinations to native targets. Since the procedure of Bremner involved Hunjara-speaking participants articulating the word forms before performing the matching task, it is likely that native target assimilation occurred and may have driven the ‘legal’ pseudoword performance we observe in the meta-analysis.

By contrast, in our own study and in Rogers and Ross (1975), participants were not instructed to articulate the test words, so nonnative elements of the word forms may have been preserved as “weird sounding.” It therefore appears that asking participants to articulate nonnative elements in a bouba/kiki test word may be a way of enhancing the ‘wordiness’ of stimuli.

## General Discussion and Conclusions

Despite the purported ubiquity of Kohler’s maluma/takete effect in the past 80 years of literature, there are surprisingly few published studies that include full data reports from ‘straight’ replications of the shape-preference method, using canonical acoustic stimuli. To put this lack of evidence in context, Köhler’s original report of the maluma/takete effect (1929/1947) includes only the comment “most people answer without hesitation,” and no more detailed statistics became available for these stimuli for 35 years, until Holland and Wertheimer’s (1964) rating task included a binary preference measure. Similarly, Ramachandran and Hubbard’s (2001) original report of the bouba/kiki effect reported 95% prevalence but omitted sample size and methodological details. Similar prevalence rates were repeated in several subsequent papers by the same authors (again, without methodological or statistical details), until they reported 98% prevalence “in a large classroom” (Ramachandran & Hubbard, 2005). The first time they reported complete experimental methods and data was in a separate experiment in 2008 (Oberman & Ramachandran, 2008) – by which time others had begun replicating the effect with more complete reporting (Maurer et al., 2006). This meta-analysis brings together results from approximately 558 participants and represents the largest collection of adult data from tests using canonical stimuli. According to this meta-analysis, future studies can expect 84% to 94% of people to give the expected response if they use pseudowords in which the majority of ‘curvy’ phonemes are /b, m, l, o, u/, and the majority of ‘spiky’ phonemes are /k, t, i, e/. Clearly, using less-canonical consonants, less-canonical vowels or a mix of congruent and incongruent phones in a single pseudoword should be expected to produce a lower response rate – as demonstrated most effectively in the graded comparisons of Fort et al. (2015) and also consistent with several other studies experimentally manipulating the degree of match (D'Onofrio, 2014; Nielsen & Rendall, 2011, 2013; Ozturk et al., 2013; Peiffer-Smadja, 2010). The findings are also consistent with the lower rates of responses observed in studies that contrast consonants that are all voiced (e.g., /m, l, n/ vs /b, d, g/, but no voiceless /p, t, k/), where congruence rates can be as low as 73% (Drijvers et al., 2015).

One possible reason that the Hunjara-speaking Songe and the Syuba show such a large departure from the published record is that failed maluma/takete experiments remain hidden away in “file drawers” of linguistics and psychology departments across the globe. If this is the case, the natural distribution of the maluma/takete effect may well be more spread, and these two data points may simply represent the unlucky few failures to detect a true effect. If so, we encourage researchers to “bring out their dead” in the interests of clarifying the scientific record. Until this happens we have to consider the alternative possibility that the data reported in the meta-analysis represent a ‘normal’ effect, observed in most populations across the globe, including the remote Himba community, and these two ‘failed’ samples differ *for other reasons*. We propose that a promising source of this ‘difference’ could be the phonetic/phontactic legality of test words in the target language, if the words are presented via audio without any articulation by the participant. We suspect that if participants cannot automatically parse linguistic strings into ordered sequences of phonemes, then well-rehearsed ancillary processes like sensory mapping may break down. This is a testable hypothesis, and future research can explicitly test whether phonetic/phonotactic legality (wordiness) is the source of the difference we observe here.

On the matter of wordiness, where sound symbolism occurs in natural languages, it is most notable in specialized words denoting mimetic onomatapoeia (e.g., *moo*), synthetic or conventionalized sound symbolism (e.g., *knock knock, sniffle*) and other kinds of words with iconic functions (Hinton, Nichols, & Ohala, 1994). While English does not have a particularly rich vocabulary for these kinds of words, some languages have a specialized word class known as ‘ideophones,’ which can be very extensive, as in the case of Japanese, with something like 4,500 onomatopoeia and ideophones (Masahiro, 2007), where sounds can be used to denote not only auditory but visual experiences (*kira kira* – ‘twinkling’), tactile textures (*sara sara* – ‘softly smooth’), bodily sensations (*peko peko* – ‘to be hungry’), manner of motions (*fuwa fuwa* – ‘floatily’) and even silence itself (*shi ∼ n*). These kinds of words are often considered ‘marked,’ in that they stand out from regular words in the language and often exhibit sound combinations or syllable patterns that are uncharacteristic of regular words in the language (Diffloth, 1979; Dingemanse, 2012). As noted by Childs (2014), the expressive nature of ideophones may result in competing demands for these parts of speech to stand out from the rest of the language while at the same time be a recognizable part of it. In his examples from Zulu, ideophones are marked by differential use of pitch (F_0_), and he suggests that suprasegmental violations (pitch) may be more acceptable than segmental (phonology, phonotactics) while still retaining inherent wordiness. The consensus therefore appears to suggest that while ideophones or expressive parts of speech *can under certain conditions* deviate from the norms of what makes a word ‘wordy,’ the ways in which ideophones deviate is also governed by its own set of regularities, as Childs terms it *constraints on violating constraints*.

Could our reduplicating /^L^bu^L^bu/ and /^H^ki^H^ki/ stimuli match a similar ‘constraint’ – a pattern for ideophones in Syuba? Syuba has a small inventory of onomatapoeic words (30 out of a lexicon of 3,339 words in one dictionary), all but two of which are explicitly mimetic (SIL, 2016). Although most of these mimetics include some degree of reduplication (e.g., /^L^tswa^H^tswã/ for the sound of a pig), none describe visual or textural characteristics of objects. Our pseudowords were presented with a pair of physical objects differing only in their edge characteristics, making it *semantically* unlikely that Syuba speakers would interpret them as novel ideophones, since Syuba does not attest this kind of onomatapoeia. However, as we did not provide a narrative frame for the unusual-sounding words, it remains possible that participants may have interpreted the unfamiliar word forms as ideophone-like (cf., Dingemanse, 2014), as noun-like, or as adjective-like. Regardless of the particular syntactic interpretation, it remains surprising that our participants show a pattern of responding that differs so dramatically from the previously reported effects of this kind.

Both languages that have so far shown this difference from the published norm share the feature that the pseudowords used for test were not word-like for the participants involved. We suspect this effect may be related to learning processes that occur early in childhood: First, ‘tuning’ to the acoustic structure of frequently heard sounds in one’s native language – a precursor to linguistically refined categorical perception of native language phonemes (Iverson & Kuhl, 1996; Kuhl, 2010; Maye, Werker, & Gerken, 2002; Werker & Tees, 1984); and second, ‘tuning’ to frequently heard sound combinations – useful for identifying word boundaries, as a precursor to word-learning (Gómez, Bootzin, & Nadel, 2006; Saffran, Aslin, & Newport, 1996). Both of these processes are normally thought of as linguistic adaptations for comprehension of ongoing meaningful speech. And yet, here we see linguistic tuning processes have created sensory effects that cascade outside purely listening for language: Linking auditory sounds to visual shapes appears not to occur systematically if the sounds are not well-represented by the (linguistically tuned) sensory processing system.

This is the first proposal in decades that attempts to explain why supposedly universal sound symbolism sometimes fails. Furthermore, it is an eminently testable hypothesis, which we hope will generate plenty of more or less successful replications, in more or less WEIRD languages. To facilitate this endeavour, you can download a template to cut out your own shapes, or 3D print them (here: https://osf.io/wt95v/). We look forward to seeing what future failures will tell us about this new theory and how our understanding of connections between visual and auditory perception can be further refined by a better understanding of the boundaries between linguistic and nonlinguistic sensation.

## Note

1 A comparison group of 24 English speakers were recruited in London. Some had acquired a second language late or in foreign language classes at school. These English speakers showed the expected pattern (congruent-to-incongruent: 22 to 2) when presented with the original testing materials and tested in English by the second author.

## References

[bibr1-2041669517724807] Best C. McRoberts G. Goodell E. (2001) American listeners’ perception of non-native consonant contrasts varying in perceptual assimilation to English phonology. Journal of Acoustical Society of America 109: 775–794. 10.1121/1.1332378 PMC2777975 11248981

[bibr2-2041669517724807] Bremner A. J. Caparos S. Davidoff J. Fockert J. D. Linnell K. J. Spence C. (2013) “Bouba” and “Kiki” in Namibia? A remote culture make similar shape–sound matches, but different shape–taste matches to Westerners. Cognition 126: 165–172. 23121711 10.1016/j.cognition.2012.09.007

[bibr3-2041669517724807] Childs G. T. (2014) Constraints on violating constraints: How languages reconcile the twin dicta of “Be different” and “Be recognizably language.”. Pragmatics and Society 5: 341–354.

[bibr4-2041669517724807] Diffloth, G. (1979). Expressive phonology and prosaic phonology in Mon Khmer. In T. L. Thongkum, et al. (Eds.), *Studies in Tai and Mon*-Khmer phonetics and phonology*: In honour of Eugenie J. A. Henderson* (pp. 49–59). Bangkok: Chulalongkorn University Press.

[bibr5-2041669517724807] Dingemanse M. (2012) Advances in the cross-linguistic study of ideophones. Language and Linguistics Compass 6: 654–672. doi:10.1002/lnc3.361.

[bibr6-2041669517724807] Dingemanse M. (2014) Making new ideophones in Siwu: Creative depiction in conversation. Pragmatics and Society 5: 384–405. doi:10.1075/ps.5.3.04din.

[bibr7-2041669517724807] D'Onofrio A. (2014) Phonetic detail and dimensionality in sound-shape correspondences: Refining the bouba-kiki paradigm. Language and Speech 57: 367–393.

[bibr8-2041669517724807] Drijvers, L., Zaadnoordijk, L., & Dingemanse, M. (2015). *Sound-symbolism is disrupted in dyslexia: Implications for the role of cross*-modal *abstraction processes.* Paper presented at the 37th Annual Meeting of the Cognitive Science Society, Austin, Texas.

[bibr9-2041669517724807] Faul F. Erdfelder E. Lang A.-G. Buchner A. (2007) G*Power 3: A flexible statistical power analysis program for the social, behavioral, and biomedical sciences. Behavior Research Methods 39: 175–191. 17695343 10.3758/bf03193146

[bibr10-2041669517724807] Fort M. Martin A. Peperkamp S. (2015) Consonants are more important than vowels in the bouba-kiki effect. Language and Speech 58: 247–266. 26677645 10.1177/0023830914534951

[bibr11-2041669517724807] Gawne L. (2013) Notes on the relationship between Yolmo and Kagate. Himalayan Linguistics 12: 1–27.

[bibr12-2041669517724807] Gawne L. (2016) My name is Maya Lama/Syuba/Hyolmo: Negotiating identity in Hyolmo diaspora communities. European Bulletin of Himalayan Research 47: 40–68.

[bibr59-2041669517724807] Gawne, L., & Styles, S. J. (2017). Cross-sensory perception for tone. OSF. doi:10.17605/OSF.IO/WT95V. Retrieved from https://osf.io/wt95v.

[bibr13-2041669517724807] Gómez R. Bootzin R. L. Nadel L. (2006) Naps promote abstraction in language-learning infants. Psychological Science 17: 670–674. 16913948 10.1111/j.1467-9280.2006.01764.x

[bibr14-2041669517724807] Gray R. Hiley R. Thom R. (2015) The sociolinguistic situation of the Hunjara-Kaina Ke [hkk] language, Oro province, Papua New Guinea (Electronic Survey Report), Dallas, TX: Summer Institute for Linguistics.

[bibr15-2041669517724807] Hackshaw A. (2009) A concise guide to clinical trials, Chicester: BMJ Books and Wiley-Blackwell.

[bibr16-2041669517724807] Henrich J. Heine S. J. Norenzayan A. (2010) The weirdest people in the world? Behavioral & Brain Sciences 33: 61–135. doi:10.1017/S0140525X0999152X. 20550733 10.1017/S0140525X0999152X

[bibr17-2041669517724807] Hinton L. Nichols J. Ohala J. J. (1994) Introduction: Sound-symbolic processes. In: Hinton L. Nichols J. Ohala J. J. (eds) Sound symbolism, Cambridge, England: Cambridge University Press, pp. 1–12.

[bibr18-2041669517724807] Holland M. K. Wertheimer M. (1964) Some physiognomic aspects of naming, or, maluma and takete revisited. Perceptual and Motor Skills 19: 111–117. 14197433 10.2466/pms.1964.19.1.111

[bibr19-2041669517724807] Hung S.-M. Styles S. J. Hsieh P.-J. (2017) Can a word sound like an object looks before you have seen it? Sound-shape mapping prior to conscious awareness. Psychological Science 28: 263–275. 28112997 10.1177/0956797616677313

[bibr20-2041669517724807] Imai M. Kita S. (2014) The sound symbolism bootstrapping hypothesis for language acquisition and language evolution. Philosophical Transactions of the Royal Society of London. Series B, Biological Sciences 369: 1–14. 10.1098/rstb.2013.0298 PMC4123677 25092666

[bibr21-2041669517724807] Iverson P. Kuhl P. K. (1996) Influences of phonetic identification and category goodness on American listeners’ perception of /r/ and /l/. Journal of the Acoustical Society of America 99: 1130–1140. 8609297 10.1121/1.415234

[bibr22-2041669517724807] Köhler W. (1929/1947) Gestalt psychology, New York, NY: Liveright Publishing.

[bibr23-2041669517724807] Kuhl P. K. (2004) Early language acquisition: Cracking the speech code. Nature Reviews Neuroscience 5: 831–843. 15496861 10.1038/nrn1533

[bibr24-2041669517724807] Kuhl P. K. (2010) Brain mechanisms in early language acquisition. Neuron 67: 713–727. 20826304 10.1016/j.neuron.2010.08.038 PMC2947444

[bibr25-2041669517724807] Ladefoged P. (1993) A course in phonetics, 3rd ed Orlando, FL: Harcourt Brace.

[bibr26-2041669517724807] Lockwood G. Dingemanse M. (2015) Iconicity in the lab: A review of behavioral, developmental, and neuroimaging research into sound-symbolism. Frontiers in Psychology 6: 1–14. 26379581 10.3389/fpsyg.2015.01246 PMC4547014

[bibr27-2041669517724807] Ludwig V. U. Adachi I. Matsuzawa T. (2011) Visuoauditory mappings between high luminance and high pitch are shared by chimpanzees (Pan troglodytes) and humans. PNAS 108: 20661–20665. 22143791 10.1073/pnas.1112605108 PMC3251154

[bibr28-2041669517724807] Masahiro, O. (Ed.). (2007). *Giongo Gitaigo 4500* [Onomatapoeia Mimetics 4500: Japanese Onomatopoeia Dictionary]. Tokyo: Shogakkan.

[bibr29-2041669517724807] Maurer D. (1993) Neonatal synesthesia: Implications for the processing of speech and faces. In: de Boysson-Bardies B. Jusczyk P. MacNeilage P. Morton J. deSchonen S. (eds) Developmental neurocognition: Speech and face processing in the first year of life, Dordrecht, the Netherlands: Kluver, pp. 109–124.

[bibr30-2041669517724807] Maurer D. Pathman T. Mondloch C. J. (2006) The shape of boubas: Sound–shape correspondences in toddlers and adults. Developmental Science 9: 316–322. 16669803 10.1111/j.1467-7687.2006.00495.x

[bibr31-2041669517724807] Maye J. Werker J. F. Gerken L. (2002) Infant sensitivity to distributional information can affect phonetic discrimination. Cognition 82: B101–B111. 11747867 10.1016/s0010-0277(01)00157-3

[bibr32-2041669517724807] Mitchell J. R. Eichentopf S. R. (2013) Sociolinguistic survey of Kagate: Language vitality and community desires, Kathmandu, Nepal: Central Department of Linguistics Tribhuvan University and SIL International.

[bibr33-2041669517724807] Möhlig W. J. G. Marten L. Kavari J. U. (2002) A grammatical sketch of Herero (Otjiherero), Köln: Cologne: Rüdiger Köppe.

[bibr34-2041669517724807] Mondloch C. Maurer D. (2004) Do small white balls squeak? Pitch-object correspondences in young children. Cognitive, Affective & Behavioral Neuroscience 4: 133–136. 10.3758/cabn.4.2.133 15460920

[bibr35-2041669517724807] Moran, S., McCloy, D., & Wright, R. (2014). *PHOIBLE Online*. Retrieved from Leipzig: Max Planck Institute for Evolutionary Anthropology: http://phoible.org/.

[bibr36-2041669517724807] Morton E. W. (1977) On the occurrence and significance of motivation-structural rules in some bird and mammal sounds. American Naturalist 111: 855–869.

[bibr37-2041669517724807] Neyeloff J. L. Fuchs S. C. Moreira L. B. (2012) Meta-analyses and forest plots using a Microsoft Excel spreadsheet: Step-by-step guide focusing on descriptive data analysis. BMC Research Notes 5: 1–6. 22264277 10.1186/1756-0500-5-52 PMC3296675

[bibr38-2041669517724807] Nielsen A. K. S. Rendall D. (2011) The sound of round: Evaluating the sound-symbolic role of consonants in the classic takete-maluma phenomenon. Canadian Journal of Experimental Psychology 65: 115–124. 21668094 10.1037/a0022268

[bibr39-2041669517724807] Nielsen A. K. S. Rendall D. (2013) Parsing the role of consonants versus vowels in the classic takete-maluma phenomenon. Canadian Journal of Experimental Psychology 67: 153–163. 23205509 10.1037/a0030553

[bibr40-2041669517724807] Oberman L. M. Ramachandran V. S. (2008) Preliminary evidence for deficits in multisensory integration in autism spectrum disorders: The mirror neuron hypothesis. Social Neuroscience 3: 348–355. doi:10.1080/17470910701563681. 18979385 10.1080/17470910701563681

[bibr41-2041669517724807] Occelli V. Esposito G. Venuti P. Arduino G. M. Zampini M. (2013) The takete–maluma phenomenon in autism spectrum disorders. Perception 42: 233–241. doi:10.1068/p7357. 23700961 10.1068/p7357

[bibr42-2041669517724807] Ohala J. J. (1994) The frequency code underlies the sound-symbolic use of voice pitch. In: Hinton L. Nichols J. Ohala J. J. (eds) Sound symbolism, Cambridge, England: Cambridge University Press, pp. 325–347.

[bibr43-2041669517724807] Ozturk O. Krehm M. Vouloumanos A. (2013) Sound symbolism in infancy: Evidence for sound–shape cross-modal correspondences in 4-month-olds. Journal of Experimental Child Psychology 114: 173–186. Retrieved from http://dx.doi.org/10.1016/j.jecp.2012.05.004. 22960203 10.1016/j.jecp.2012.05.004

[bibr44-2041669517724807] Peiffer-Smadja, N. (2010). *Exploring the bouba/kiki effect: A behavioral and fMRI study* (Master Recherche en Sciences Cognitives Master Thesis). Université Paris Descartes, Paris.

[bibr45-2041669517724807] Polka L. Bohn O.-S. (2003) Asymmetries in vowel perception. Speech Communication 41: 221–231.

[bibr46-2041669517724807] Ramachandran V. S. Hubbard E. M. (2001) Synaesthesia—A window into perception, thought and language. Journal of Consciousness Studies 8: 3–34.

[bibr47-2041669517724807] Ramachandran V. S. Hubbard E. M. (2005) The emergence of the human mind: Some clues from synesthesia. In: Robertson L. C. Sagiv N. (eds) Synesthesia: Perspectives from cognitive neuroscience, Oxford, England: Oxford University Press, pp. 147–190.

[bibr48-2041669517724807] Rogers S. K. Ross A. S. (1975) A cross-cultural test of the maluma-takete phenomenon. Perception 4: 105–106. 1161435 10.1068/p040105

[bibr49-2041669517724807] Saffran J. R. Aslin R. N. Newport E. L. (1996) Statistical learning by 8-month old infants. Science 274: 1926–1928. 8943209 10.1126/science.274.5294.1926

[bibr50-2041669517724807] Schwartz J.-L. Abry C. Boë L.-J. Ménard L. Vallée N. (2005) Asymmetries in vowel perception, in the context of the dispersion–focalisation theory. Speech Communication 45: 425–434.

[bibr51-2041669517724807] SIL (Producer). (2016). *Syuba Webonary*. Retrieved from http://syuba.webonary.org/?s=&search=Search&key=&tax=15056&lang=en.

[bibr52-2041669517724807] Spence C. (2011) Crossmodal correspondences: A tutorial review. Attention, Perception and Psychophysics 73: 971–995. 10.3758/s13414-010-0073-7 21264748

[bibr53-2041669517724807] Styles, S. J. (2014). *Sound symbolism is not universal: A phonological/tonological basis for matching speech-sounds to shapes*. Paper presented at the International Multisensory Research Forum, Amsterdam.

[bibr54-2041669517724807] Teo, A., Gawne, L., & Baese-Berk, M. (2015). *Tone and intonation: A case study in two Tibetic languages.* Paper presented at the 18th International Conference on Phonetic Sciences, Glasgow.

[bibr55-2041669517724807] Walker P. Bremner J. G. Mason U. Spring J. Mattock K. Slater A. Johnson S. P. (2010) Preverbal infants’ sensitivity to synaesthetic cross-modality correspondences. Psychological Science 21: 21–25. 20424017 10.1177/0956797609354734

[bibr56-2041669517724807] Werker J. F. Tees R. C. (1984) Cross-language speech perception: Evidence for perceptual reorganization during the first year of life. Infant Behavior and Development 7: 49–63.

[bibr57-2041669517724807] Westbury C. (2005) Implicit sound symbolism in lexical access: Evidence from an interference task. Brain & Language 93: 10–19. 15766764 10.1016/j.bandl.2004.07.006

[bibr58-2041669517724807] Zipf G. K. (1935) The psychobiology of human language, Boston, MA: Houghton-Mifflin.

